# Molecular and morphological surface analysis: effect of filling pastes and cleaning agents on root dentin

**DOI:** 10.1590/1678-77572016-0053

**Published:** 2017

**Authors:** Vanessa Benetello DAINEZI, Alexsandra Shizue IWAMOTO, Airton Abrahão MARTIN, Luís Eduardo Silva SOARES, Yumiko HOSOYA, Fernanda Miori PASCON, Regina Maria PUPPIN-RONTANI

**Affiliations:** 1 Universidade Estadual de Campinas, Faculdade de Odontologia de Piracicaba, Departamento de Odontopediatria, Piracicaba, SP, Brasil.; 2 Universidade do Vale do Paraíba, Univap, Faculdade de Ciências da Saúde, Odontologia, São José dos Campos, SP, Brasil, São José dos Campos, SP, Brasil.; 3 Tohoku University Graduate School of Dentistry, Division of Pediatric Dentistry Department of Oral Health and Development Sciences, Sendai, Japan.; 4 Universidade Federal do Piauí, Campus Ministro Petrônio Portella, Departamento de Física , CCN Bairro Ininga, Teresina, PI, Brasil.

**Keywords:** Deciduous tooth, Root canal filling materials, Raman spectroscopy, X-Ray Emission Spectrometry, Scanning electron microscopy

## Abstract

The quality of the dentin root is the most important factor for restoration resin sealing and drives the outcome of endodontic treatment.

**Objective:**

This study evaluated the effect of different filling pastes and cleaning agents on the root dentin of primary teeth using Fourier-transformed Raman spectroscopy (FT-Raman), micro energy-dispersive X-ray fluorescence (µ-EDXRF) and scanning electron microscopic (SEM) analysis.

**Material and Methods:**

Eighty roots of primary teeth were endodontically prepared and distributed into 4 groups and filled according to the following filling pastes: Control-no filling (CP), Calen^®^+zinc oxide (CZ), Calcipex II^®^ (CII), Vitapex^®^ (V). After seven days, filling paste groups were distributed to 4 subgroups according to cleaning agents (n=5): Control-no cleaning (C), Ethanol (E), Tergenform^®^ (T), 35% Phosphoric acid (PA). Then, the roots were sectioned and the dentin root sections were internally evaluated by FT-Raman, µ-EDXRF and SEM. Data was submitted to two-way ANOVA and Tukey tests (α=0.05).

**Results:**

Regarding filling pastes, there was no significant difference in organic content. CP provided the lowest calcium values and, calcium/phosphoric ratio (Ca/P), and the highest phosphoric values. For cleaning agents there was no difference in organic content when compared to the C; however, T showed significantly higher calcium and Ca/P than PA. All groups showed similar results for phosphorus. The dentin smear layer was present after use of the cleaning agents, except PA.

**Conclusion:**

The filling pastes changed the inorganic content, however they did not change the organic content. Cleaning agents did not alter the inorganic and organic content. PA cleaned and opened dentin tubules.

## Introduction

After root canal treatment of primary teeth, the tooth can be restored with an adhesive material, which seals the tooth from microleakage^22^. However, the filling pastes used in Pediatric Dentistry could interfere with final restoration resin and its adhesion to dentin. The composition of the materials, such as oily excipient and calcium hydroxide, would act as contaminants and decrease bonding strength in dentin^9,12^. In addition, the adhesion technique requires infiltrated monomers polymerized in clean dentin surface, for a successful retention of adhesive restoration^15^. Thus, in order to obtain satisfactory restoration longevity, the dentin surface has to be free of debris and with ideal features to allow for an adequate hybridization of the resin monomers and dentin^5^. The quality of the root filling and the restoration sealing are the most important factors for the endodontic treatment’s outcome^6^. Thus, the cementation of fiber posts, which are used on severely damaged primary anterior teeth need to bond efficiently to the composite resin.

Calcium hydroxide’s effect on the physical and sealing properties of canal sealers was investigated^10^. It was observed that Calcipex^®^, a water-based filling paste, composed of calcium hydroxide, barium sulfate and distilled water was easy to handle, and also easily removed^10^. In addition, Vitapex^®^, a silicon oil-based, composed of calcium hydroxide, iodoform and silicone oil was sticky and adhered to large areas of the root canal wall, resulting in significant amounts of residual paste after cleaning the root surface. Therefore, it is supposed that Vitapex^®^ and Calcipex^®^ when used as filling pastes have different levels of penetration and stickiness on the dentin surface, and would be easily removed depending on the excipient contained.

In order to clean and prepare the root canal surface for dentin bonding, cleaning agents can be used to remove the residual filling paste from the dentin. An anionic detergent solution (sodium diethylene glycol lauryl ether sulfate) can be used to clean the dentin surface, since it reduces the superficial tension of liquids and suspension of the debris that results from root preparation. In addition, it could also adequately work for oil removal^14^. Ethanol is another cleaning agent that can be used to remove some oil and polyethylene glycol based filling pastes, since it is an organic solvent^26^. The use of ethanol on dentin promotes drying, inducing a hydrophobic dentin surface, an advantage that allows for the infiltration of resin monomers to wet dentin, increasing resin retention^23^.

Phosphoric acid used as an etching agent is used in etch & rinse adhesive systems, despite demineralizing the substrate surface during adhesive procedures, it can also Benetello considered as a surface cleaning agent that can clean dentin, since it removes the smear layer, superficially demineralizes inter and peritubular dentin and exposes the collagen matrix without changing the intrinsic characteristics of the inorganic dentin content^2,3^.

In addition, the dentin surface, after cleaning filling pastes residues, can be modified by the various steps of the canal preparation and cleaning procedures. It is known that the simple use of saline solution can modify the dentin surface^18^ and can jeopardize the bonding strength of adhesive restorative materials, compromising the endodontic treatment’s success.

When using the filling pastes and the cleaning agents, the evaluation of organic and inorganic content of dentin is an important factor for observing alterations in the dentin and could be helpful for selecting the best cleaning agent to be used in filled roots, without reducing adhesion. The calcium hydroxide-based pastes can alter root dentin properties such as hardness and modulus of elasticity^29^. In addition, depending on size and shape, the calcium hydroxide particles can penetrate into the dentin tubules^11^, occluding them and be detrimental to adhesion. On the other hand, cleaning agents, such as ethanol, can expand and maintain collagen fibrils of dentin^20^. Therefore, anionic detergent solution removed the smear layer and opened the dentin tubule and uncovered collagen fibrils^17^; its use combined with phosphoric acid demineralizes the dentin, which could modify both the organic and the inorganic content^16^.

Therefore, the dentin structure and composition can be evaluated through sensitive analysis of the dentin’s chemical aspects to determine inorganic and organic modifications. In this context, Fourier-transformed Raman spectroscopy (FT-Raman) and micro energy-dispersive X-ray fluorescence (µ-EDXRF ) are established methods for simple and nondestructive analyses for obtaining information with respect to molecular composition and substrate structure. µ-EDXRF allows for the identification and quantification of dentin minerals such as calcium (Ca) and phosphorus (P)^24,25^, and FT-Raman analyses in the 2940-2942 cm^-1^ band (C-H stretching) of the organic content^1-3,13^. Also, the scanning electron microscopic (SEM) can be used to observe details, post-treatment, of the root canal dentin surface of primary teeth.

Thus, the aim of this *in vitro* study was to verify the effect of different filling pastes and cleaning agents on primary teeth root dentin. The hypothesis tested was that different filling pastes, associated with different cleaning agents, affect the molecular and surface features of the root canal dentin surface of primary teeth.

## Material and methods

This study was conducted after receiving approval from The Ethics Committee in Research of the Dental School.

### Sample selection

Eighty extracted, human anterior primary teeth were cleaned with saline solution to remove any remaining debris and soft tissue. The samples were frozen and stored for no more than 6 months until analysis. The selection criteria included those with at least two thirds of the root length and with no prior exposure to endodontic therapy.

### Specimen preparation

The crowns were sectioned at the cementoenamel junction (CEJ) using a high-speed diamond saw with water-cooling and were subsequently discarded. Then, the roots were sectioned longitudinally, and only one slice from each root was selected, 80 specimens were obtained. The specimens were selected under a stereo microscope and those that showed cracks or other structural alterations were excluded. Thereafter, the lateral walls of each specimen were ground flat with 320-grit SiC paper, and sonicated for 60 seconds with deionized water to remove the residues from the lumen of the root canal of primary teeth.

The specimens were prepared to a working length until the apex using K-ﬁles (#15-35 sizes) (Dentsply Maillefer, Baillagues, Switzerland; batch number 8313220), simulating the clinical preparation of a root canal and working at the lumen of the root canal. Between each ﬁling, the lumen of the canals were irrigated with 2 mL of 0.5% NaOCl solution (RioLab, Brazil; batch number CH95436). During preparation, EndoPTC (Biodinâmica Química e Farmacêutica Ltda., Ibiporã, PR, Brazil; batch number 83510) + 0.5% NaOCl solution was used^20^. The canals were dried with paper points.

The specimens were distributed into 4 groups (n=20) according to the filling paste: Control (CP) (no filling); Calen^®^ paste thickened zinc oxide (CZ) (1.0 g:1.0 g); Calcipex II^®^ (CII); Vitapex^®^ (V). The CZ paste was placed onto the lumen of the primary teeth root canal using a K-file; CII and V were placed directly on the lumen of the root canal with a pre-packed syringe (Figure 1A). Then, specimens were stored at a relative humidity of 100% at 37°C for seven days so as to simulate a clinical procedure. Next, all the filling pastes visible on the lumen of the root canal were removed with the aid of curettes and K-files. Subsequently, each group was distributed into 4 groups (n=5) according to cleaning agents: control group (C) (no cleaning agent); Ethanol (E): a cotton wrapped file was soaked in 70% ethanol for 10 seconds and rubbed onto the lumen of the root canal; Tergenform^®^ (T): a cotton wrapped file was soaked in T for 60 seconds and rubbed onto the lumen of the root canal; Phosphoric acid (PA): the lumen of the root canal was etched with 35% phosphoric acid for 15 seconds and later rinsed under water for 30 seconds. All specimens were dried with paper points. After that, they were stored at a relative humidity of 100% at 37°C until the molecular and morphological analyses were conducted (Figure 1B). The application times of E and T were established in a pilot study. All materials used (including manufacturer details) are described in Figure 2.

**Figure 1A:**
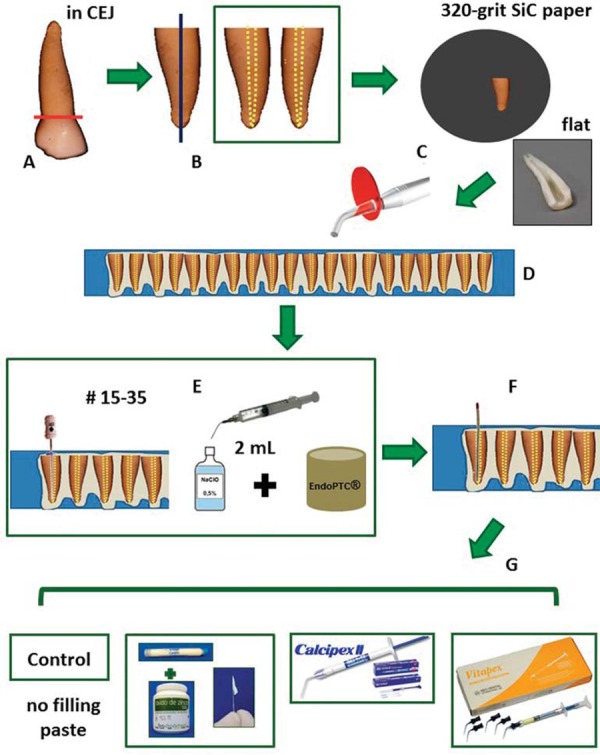
Material and methods. (A) the crowns were sectioned at the cementoenamel junction (CEJ) and discarded; (B) the roots were sectioned longitudinally; (C) the lateral walls were ground flat and sonicated to remove the residues from the lumen of the primary teeth root canal; (D) the specimens were fixed in composite resin, the lumen was without contact with resin; (E) the lumen of the root canal was prepared to a working length until the apex using K-ﬁles (#15-35 sizes). Between each ﬁle the lumen of the canals was irrigated with 2 mL of 0.5% NaOCl solution. During preparation, EndoPTC + 0.5% NaOCl solution was used; (F) the canals were dried with paper points; (G) the specimens were distributed into 4 groups (n=20) according to the filling paste: Control-CP (no filling); Calen® paste thickened zinc oxide-CZ (1.0 g:1.0 g); Calcipex II®-CII; Vitapex®-V

**Figure 1B:**
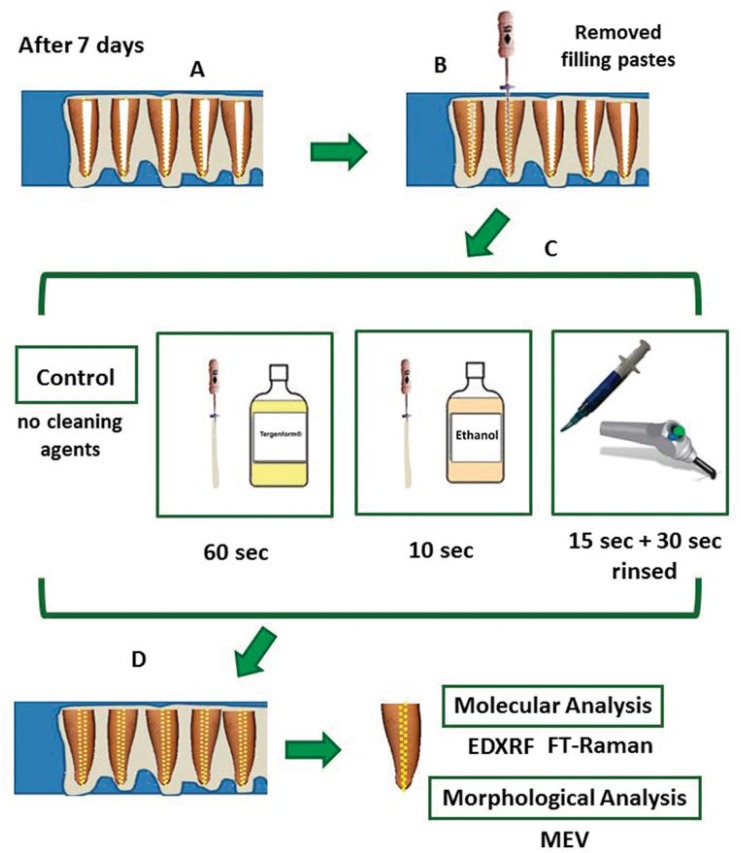
Material and methods. (A) specimens were stored at a relative humidity of 100% at 37°C for seven days; (B) all the filling pastes which were visible on the lumen of the root canal were removed with the aid of curettes and K-files; (C) each group was distributed into 4 groups (n=5) according to cleaning agents: control group-C (no cleaning agent); Ethanol-E: for 10 seconds; Tergenform®-T: for 60 seconds; Phosphoric acid-PA: 35% phosphoric acid for 15 seconds and later rinsed under water for 30 seconds; (D) the specimens were stored at a relative humidity of 100% at 37°C until the molecular and morphological analyses were conducted.

### Molecular analysis

The cervical third of each lumen of the root canal was analyzed by Fourier-transformed Raman spectroscopy (FT-Raman) and µ-EDXRF spectroscopy in order to evaluate changes in the dentin’s organic and inorganic components, respectively. Both analytical methods were nondestructive and the data collection was based on laser or X-ray interactions with the sample, without contact.

### FT-Raman spectroscopic analysis

Spectra of the samples were obtained using an FT-Raman Spectrometer (RFS 100/S; Bruker Inc. Karlsruhe, Germany). To excite the spectra, the defocused 1064.1 nm line of an Nd:YAG laser source was used. The maximum incident laser power on the sample surface was approximately 150 mW and the spectrum resolution was 4 cm^-1^.

The samples were positioned in the sample holder compartment and an IR352 lens collected radiation scattered through 90° on the dentin surface. For each sample, one spectrum was collected at a central point on the cervical dentin root. In order to obtain a good signal to noise ratio, 100 scans were co-added for each spectra. Five spectra were obtained in each group.

The changes in the organic dentin components were analyzed by comparing the integrated areas of the Raman peak centered at 2940 cm^1^. The integrated areas of the peaks were calculated with the software Microcal Origin 6.0 (Microcal Software, Inc., Northampton, MA, USA).

### Micro energy-dispersive X-ray fluorescence analysis

Semi-quantitative elemental analyses of calcium (Ca) and phosphorus (P) were carried out with an energy-dispersive micro X-ray ﬂuorescence spectrometer (model µEDX 1300; Shimadzu, Kyoto, Japan) equipped with a rhodium X-ray tube and a liquid nitrogen (N_2_) cooled, Si(Li) detector and coupled to a computer system for data processing. The voltage in the tube was set at 15 kV, with automatic adjustment of the current. Three spectra from each specimen were collected after the treatments. The measurements were performed with a count rate of 100 sec. *per* point. The equipment calibration and the chemical balance were performed as previously reported^25^.

In addition, the filling pastes were analyzed by chemical elemental analysis using X-ray ﬂuorescence (µ-EDXRF ). A portion of 0.1 g of the filling pastes was analyzed by µ-EDXRF using three points.

### Morphological analysis

#### Scanning electron microscopic (SEM) analysis

The specimens were dried and mounted on a holder using double-sided adhesive carbon tape. The samples were sputter-coated with gold (Balzers-SCD 050 Sputter Coater, Liechtenstein) and examined with a scanning electron microscope (JEOL JSM 5600 LV, Tokyo, Japan) operating at 1000x magnifications. The morphological analysis was described according to the images obtained by SEM.

## Statistical analysis

All data obtained is expressed in average and standard deviations. Data was submitted to the Lilliefors test for normality and only for the Ca/P data log transformation was used. The study factors were filling pastes and cleaning agents, statistical analysis was performed by two-way ANOVA followed by Tukey’s test using Assistat 7.6 beta (Campina Grande, PB, Brazil), for both FT-Raman and µ-EDXRF , and the significance level was set at 5%.

## Results

According to the two-way ANOVA statistical analysis applied to the FT-Raman and µ-EDXRF , there was no interaction between the studied factors (filling pastes and cleaning agents) (p>0.05). Considering the filling pastes in the FT-Raman analysis (2940 cm^-1^ band), no significant difference was found between filling pastes (p≥0.05). Therefore, filling pastes did not produce any change in the organic content of the dentin. (Table 1)

**Table 1:** Mean and standard deviations of calcium (wt %), phosphorus (wt %) and Ca/P ratio concerning X-ray ﬂuorescence analysis, and FT-Raman spectroscopic analysis for peak area (2940 cm-1) in primary root dentin for the filling pastes

Pastes	EDXRF			FT- Raman
	Ca	P	Ca/P	2940 cm^**-1**^
Control (no filling)	21.57±0.82^b^	12.15±0.73^a^	1.77±0.04^b^	1.08±0.14^a^
Calen^®^ + Zinc Oxide -CZ	23.71±1.08^a^	10.77±0.76^b^	2.22±0.12^a^	1.06±0.13^a^
Calcipex II^®^-CII	23.51±1.04^a^	9.98±0.81^b^	2.41±0.27^a^	1.04±0.10^a^
Vitapex^®^	24.15±1.63^a^	10.80±0.44^b^	2.25±0.16^a^	1.04±0.09^a^

A lowercase letter following a value indicates signiﬁcant differences between groups

Considering the inorganic content of the filling paste (Table 1), µ-EDXRF analysis showed that the Ca content and Ca/P ratio of the control group (no filling) had significantly lower values. For the P content the opposite was verified, and the control group (no filling) showed higher P values. However, the filling paste groups (CZ, CII and V) showed no significant difference between them for Ca, P and Ca/P (p≥0.05).

Regarding cleaning agents (Table 2), concerning the FT-Raman analysis, T showed significantly higher organic content than the ethanol group (p<0.05). In µ-EDXRF, T showed higher values for Ca content and Ca/P ratio than the phosphoric acid group. All groups were similar for P content with regards to the µ-EDXRF analysis.

**Table 2:** Mean and standard deviations of calcium (wt %), phosphorus (wt %) and Ca/P ratio with regards to X-ray ﬂuorescence analysis and FT-Raman spectroscopic analysis for peak area (2940 cm-1) in primary root dentin for the cleaning agents

Cleaning Agents	EDXRF			FT- Raman
	Ca	P	Ca/P	2940 cm^**-1**^
Control (no cleaning)	22.97±2.10^ab^	10.65±1.18^a^	2.20±0.30^ab^	1.05±0.06^ab^
Ethanol - E	23.58 ±1.05^ab^	10.92±1.20^a^	2.20±0.31^ab^	0.94±0.05^b^
Tergenform^®^- T	24.06±1.13^a^	10.87±1.45^a^	2.28±0.40^a^	1.18±0.06^a^
Phosphoric Acid - PA	22.33±1.33^b^	11.26±1.20^a^	1.99±0.10^b^	1.04±0.08^ab^

A lowercase letter following a value indicates signiﬁcant differences between groups

The chemical elements of the filling pastes identified and quantified by µ-EDXRF are shown in Figure 3 and Table 3, respectively. Table 4 shows that all filling pastes contained calcium in their composition. The CZ filling paste also contained zinc in a proportion higher than calcium. The CII filling pastes contained barium, titanium and sulfur, but had a higher percentage of calcium. The V filling paste contained iodine and silicon in higher proportions than calcium.

**Figure 2:**
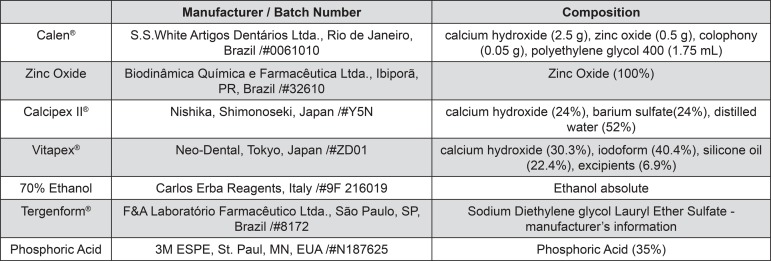
Details of the materials

**Table 3:** Results obtained by X-ray fluorescence analysis show the chemical elements silicon (Si), sulfur (S), calcium (Ca), titanium (Ti), zinc (Zn), iodine (I), barium (Ba) identified in filling pastes as percentages

Elements	Calen^**®**^ + Zinc Oxide	Calcipex II^**®**^	Vitapex^**®**^
	**(%)**	**(%)**	**(%)**
Si	-	-	42.29
S	-	8.14	-
Ca	26.79	40.48	27.91
Ti	-	12.46	-
Zn	73.21	-	-
I	-	-	29.80
Ba	-	38.92	-

Morphological analysis of the root dentin of primary teeth surfaces using SEM showed retention of filling paste amount and smear layers upon the dentin surface for all cleaning agent groups, except when PA was used, which completely removed the filling pastes on the dentin surface (Figure 4). For the control group (no filling) and no clean up or clean up with E or T, similar characteristics of the dentin surface were found: dentin tubules and dentin surface covered by dense smear layer, whereas cleaned up with PA dentin surface of root canal filled with different filling pastes showed intertubular dentin with a corroded aspect, visible dentin tubules and peritubular dentin demineralized with widespread tubule entrance. The other groups, otherwise, showed some areas with less corroded intertubular dentin, however, they were all cleaned up by the filling pastes. Apparently, specimens filled up with V showed remaining debris even after using PA. Overall, all filling pastes, E and T, used in this study, did not show any ability to remove the smear layer and showed a dirty surface with filling paste debris remaining on the dentin surface.

**Figure 3:**
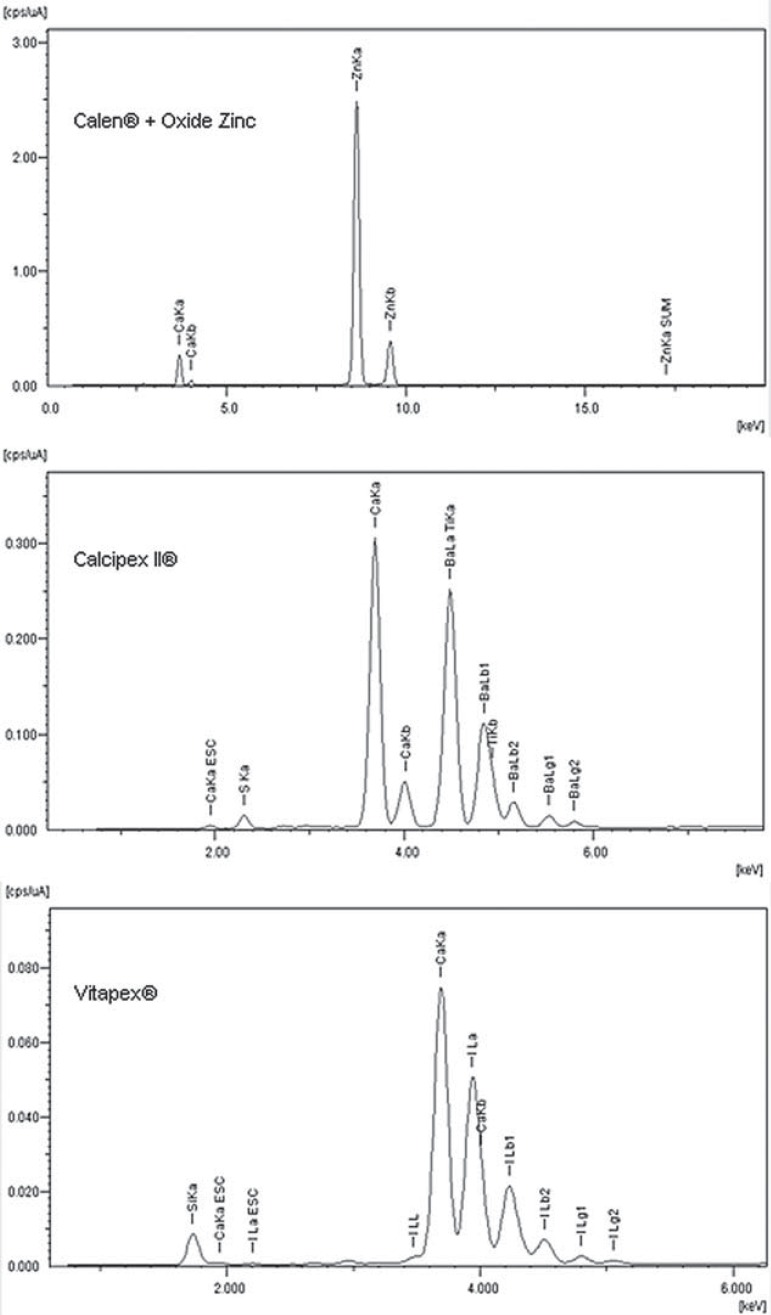
Chemical elements spectra silicon (Si), sulfur (S), calcium (Ca), titanium (Ti), zinc (Zn), iodine (I) and barium (Ba) showed by X-ray fluorescence analysis line-scan in the filling pastes groups

## Discussion

The hypothesis tested, in which different filling pastes associated with different cleaning agents affect the molecular and surface features of the root canal dentin surface of primary teeth was not accepted. Regarding the FT-Raman and µ-EDXRF, there was no interaction between the studied factors (filling pastes and cleaning agents).

There were significant differences in the inorganic content regarding Ca, P and Ca/P for the µ-EDXRF analysis when comparing all filling paste groups (Table 1). For Ca and Ca/P, the values (%w) were higher for the filling paste groups when compared to the control group (no filling), and for P the values were lower than the control group (no filling). In addition, between the filling pastes there were no significant differences. However, there was also no significant difference in the organic content of dentin when FT-Raman analysis was conducted (Table 1). The increased Ca values could be explained given that all the tested filling pastes are calcium-based (Table 3) and they remained on dentin surface associated with the smear layer. However, based on the microanalysis of chemical containing pastes, it was noticed that the amount of Ca in the filling pastes is not essential for increasing the Ca on dentin, since CII presented the highest Ca (Table 3) value and it did not provide a higher Ca %w (Table 1) available on the dentin when compared with the other filling pastes. On the other hand, the P content was higher in the control group (no filling) compared to the filling paste groups. It could be observed that the filling pastes that remained on the dentin surface over the smear layer probably blocked or prohibited phosphate analysis. Similar results were described by Rythén, et al.^21^ (2010), in which exfoliated primary teeth showed higher Ca than P on the dentin, the study was performed using X-ray microanalyses.

In addition, there was no significant difference in the organic content (FT- Raman) when the cleaning agents were compared to the control (no cleaning agent), however, the root dentin of primary teeth treated with T showed higher amounts of collagen than those treated with E (Table 2). The 2940-2942 cm^-1^ band (C-H stretching) was used to semi-quantify the relative organic changes, since it is sharper and stronger than the 1254 and 1667cm^-1^ amide bands^1-3,13^.

This study showed that since the filling pastes used as cleaning solutions did not provide changes in collagen related to control groups, it seems that there was no significant alteration when considering the organic content. This is clinically important, because dentin is composed of, 20% in weight^8^ or 30% in volume^8^, hydrated organic matrix, most of which consists of type I collagen. It constitutes ~90% of the organic matrix^8^. This can be good for bonding since even after filling, in an endodontic treatment, or when using those cleaning agents, dentin collagen was not modified. Ideally, this layer/zone is a structurally integrated resin-collagen biopolymer hybrid that provides a continuous and durable link between the bulk adhesive and dentin substrate^30^. The result of this study corroborates with Borges, et al.^1^ (2007) and Borges, et al.^2^ (2008) showing that no alterations were found in the organic content of primary teeth dentin when an acid etch procedure was used.

The root dentin of primary teeth cleaned with T showed contents of Ca and a Ca/P ratio higher than PA and did not show significant differences between the other groups. T is a detergent with an alkaline pH, which did not show dentin demineralization nor remove the filling pastes, even after cleaning. Although there was a difference between them, they were similar to the control group (no cleaning agent). PA was similar to the control group (no cleaning agent), probably due to the higher calcium levels in the filling pastes, which would buffer phosphoric acid, an effect associated with increasing pH, and the demineralization rate decreased in those groups^28^, with no time interference^4^. Another explanation or additional effect would be that the high carbonate content in primary teeth dentin is inversely proportional to the calcium content^27^, which probably contributed or added to the buffering effect. In addition, regarding the buffering effect, while acid etching removes up to 30% of the calcium phosphates, it dissolves 75% of the carbonates^7^. Therefore, the calcium phosphate was less affected, while the carbonate was removed, showing that the Ca% was similar to the etched PA and the control groups (no cleaning agent).

**Figure 4:**
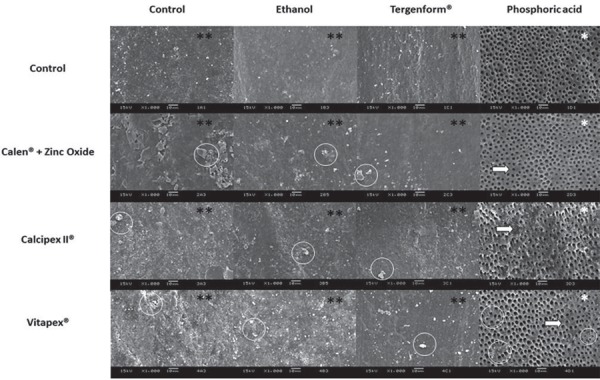
Representative scanning electron microscopy (SEM) images of root dentin surfaces of primary teeth, regarding the pastes and cleaning agents (original magnifications: 1000x). SEM observations revealed the presence of a heavy smear layer (**) in all groups. For the phosphoric acid group, no smear layer was shown, and all tubules were cleaned and opened (*). White circles represent particles of filling pastes remaining on the dentin surface. White arrows point out non-demineralized intertubular dentin areas. White circles signify filling paste residues

Representative SEM images (Figure 4) of the root dentin surfaces of primary teeth showed clean and open tubules when all filling paste groups were used and were etched by PA. Borges, et al.^2^(2008) also showed that primary teeth dentin etched by phosphoric acid enhanced the tubules ability to open and enlarge. In addition, the images of the groups that used filling pastes and phosphoric acid showed some areas of non-demineralized intertubular dentin. Studies showed that after use of calcium hydroxide dressings on the root canal and cleaning there is residual of calcium hydroxide that causes decreases to the bond strength^9,12^ and even after the use of phosphoric acid there was no increase in the bond strength^12^. However, other cleaning agents studied showed tubules recovered by a heavy smear layer and filling paste debris. To our knowledge, there have been no reports that verified the molecular and morphological features of the root dentin of primary teeth when filling pastes and cleaning agents were used.

The results from this study provided important information concerning the effect of root canal filling pastes used in Pediatric Dentistry and the possible cleaning agents used to remove these filling pastes in order to enhance the restorative procedure. In fact, it could be observed that phosphoric acid used on the dentin surface of the root canal can clean and provide an adequate surface for bonding, as it is commonly known that effective adhesion technique requires the infiltrated monomers that are polymerized in cleaning dentin^15^. The results obtained from this study are promising, since it does not use up any cleansing agent, but uses two-step etching and adhesive rinsing, the adhesion of restorative materials is feasible, since it is less time consuming. In addition, the use of a self-etching system can be used with E or T or PA, which should be the best choice to cleaning the root dentin surfaces of primary teeth, regardless of filling paste, since, they did not change the inorganic or organic content and also allowed for a smear layer on the dentin surface.

## Conclusion

Based on our findings, regardless of filling pastes, all cleaning agents, in spite of providing some disturbance on the dentin surface, cannot be considered as harmful for cleaning the root dentin. The filling pastes and cleaning agents studied did not alter the organic content of primary teeth root dentin. The inorganic content (Ca, P and Ca/P) on the root dentin of primary teeth was altered by filling pastes, showing a residual mineral amount; however, cleaning agents did not change this. Morphological features of the root dentin of primary teeth were affected by phosphoric acid group treatment, showing cleaned and open dentin tubules.
